# GLIO-Select: Machine Learning-Based Feature Selection and Weighting of Tissue and Serum Proteomic and Metabolomic Data Uncovers Sex Differences in Glioblastoma

**DOI:** 10.3390/ijms26094339

**Published:** 2025-05-02

**Authors:** Erdal Tasci, Shreya Chappidi, Ying Zhuge, Longze Zhang, Theresa Cooley Zgela, Mary Sproull, Megan Mackey, Kevin Camphausen, Andra Valentina Krauze

**Affiliations:** Radiation Oncology Branch, Center for Cancer Research, National Cancer Institute, NIH, 9000 Rockville Pike, Building 10, Bethesda, MD 20892, USA; erdal.tasci@nih.gov (E.T.); shreya.chappidi@nih.gov (S.C.); zhugey@mail.nih.gov (Y.Z.); longze.zhang@nih.gov (L.Z.); theresa.cooleyzgela@nih.gov (T.C.Z.); sproullm@mail.nih.gov (M.S.); mmackey@mail.nih.gov (M.M.); camphauk@mail.nih.gov (K.C.)

**Keywords:** glioblastoma, biological sex differences, omics, proteomic, metabolomic, feature selection, machine learning, pattern recognition

## Abstract

Glioblastoma (GBM) is a fatal brain cancer known for its rapid and aggressive growth, with some studies indicating that females may have better survival outcomes compared to males. While sex differences in GBM have been observed, the underlying biological mechanisms remain poorly understood. Feature selection can lead to the identification of discriminative key biomarkers by reducing dimensionality from high-dimensional medical datasets to improve machine learning model performance, explainability, and interpretability. Feature selection can uncover unique sex-specific biomarkers, determinants, and molecular profiles in patients with GBM. We analyzed high-dimensional proteomic and metabolomic profiles from serum biospecimens obtained from 109 patients with pathology-proven glioblastoma (GBM) on NIH IRB-approved protocols with full clinical annotation (local dataset). Serum proteomic analysis was performed using Somalogic aptamer-based technology (measuring 7289 proteins) and serum metabolome analysis using the University of Florida’s SECIM (Southeast Center for Integrated Metabolomics) platform (measuring 6015 metabolites). Machine learning-based feature selection was employed to identify proteins and metabolites associated with male and female labels in high-dimensional datasets. Results were compared to publicly available proteomic and metabolomic datasets (CPTAC and TCGA) using the same methodology and TCGA data previously structured for glioma grading. Employing a machine learning-based and hybrid feature selection approach, utilizing both LASSO and mRMR, in conjunction with a rank-based weighting method (i.e., GLIO-Select), we linked proteomic and metabolomic data to clinical data for the purposes of feature reduction to identify molecular biomarkers associated with biological sex in patients with GBM and used a separate TCGA set to explore possible linkages between biological sex and mutations associated with tumor grading. Serum proteomic and metabolomic data identified several hundred features that were associated with the male/female class label in the GBM datasets. Using the local serum-based dataset of 109 patients, 17 features (100% ACC) and 16 features (92% ACC) were identified for the proteomic and metabolomic datasets, respectively. Using the CPTAC tissue-based dataset (8828 proteomic and 59 metabolomic features), 5 features (99% ACC) and 13 features (80% ACC) were identified for the proteomic and metabolomic datasets, respectively. The proteomic data serum or tissue (CPTAC) achieved the highest accuracy rates (100% and 99%, respectively), followed by serum metabolome and tissue metabolome. The local serum data yielded several clinically known features (PSA, PZP, HCG, and FSH) which were distinct from CPTAC tissue data (RPS4Y1 and DDX3Y), both providing methodological validation, with PZP and defensins (DEFA3 and DEFB4A) representing shared proteomic features between serum and tissue. Metabolomic features shared between serum and tissue were homocysteine and pantothenic acid. Several signals emerged that are known to be associated with glioma or GBM but not previously known to be associated with biological sex, requiring further research, as well as several novel signals that were previously not linked to either biological sex or glioma. EGFR, FAT4, and BCOR were the three features associated with 64% ACC using the TCGA glioma grading set. GLIO-Select shows remarkable results in reducing feature dimensionality when different types of datasets (e.g., serum and tissue-based) were used for our analyses. The proposed approach successfully reduced relevant features to less than twenty biomarkers for each GBM dataset. Serum biospecimens appear to be highly effective for identifying biologically relevant sex differences in GBM. These findings suggest that serum-based noninvasive biospecimen-based analyses may provide more accurate and clinically detailed insights into sex as a biological variable (SABV) as compared to other biospecimens, with several signals linking sex differences and glioma pathology via immune response, amino acid metabolism, and cancer hallmark signals requiring further research. Our results underscore the importance of biospecimen choice and feature selection in enhancing the interpretation of omics data for understanding sex-based differences in GBM. This discovery holds significant potential for enhancing personalized treatment plans and patient outcomes.

## 1. Introduction

Glioblastoma (GBM) is a fatal primary brain tumor that affects men disproportionately compared to women (1.6:1), with data suggesting that males have more aggressive diseases and poorer outcomes [1]. The biological reasons for these observations are as yet unclear, although molecular characterization reveals that females are more likely to have MGMT methylated disease, secondary GBMs, differences in tumor location, and molecular and metabolic mechanistic triggers [2,3,4,5,6]. Sex differences in cancer as applied to serum proteomics and metabolomics are important given mounting data for the differential response to management and outcome differences between male and female patients. This is particularly the case in non-invasively collected biospecimens such as serum where molecular profiling aimed at sex differences is underexplored [1,6,7,8,9,10,11]. Nonetheless, non-invasive specimens are easier and cheaper to acquire and provide the only means of obtaining data in real-time in tumors located in areas not amenable to repeat tissue sampling, such as the brain. Noninvasive biospecimens such as blood samples in the clinic are the mainstay of biomarker-directed clinical care in the real world and the most cost and time-effective means of obtaining information that can impact management in real-time. Blood-based biomarkers are not currently available for most cancers including GBM, limiting the ability to personalize management and curtailing access to precision medicine secondary to cost, particularly in resource-strained care environments [12,13]. Sex as a biological variable analysis (SABV) has been hampered by a paucity of data available with robust annotation for male and female as class labels and the prevalence of datasets where one sex is more frequently diagnosed with the primary tumor as compared to the other, as is the case for GBM [14]. Most analyses in this space employ data as an aggregate of both men and women without separation of the samples for analysis, masking potential differences. Additional barriers in serum proteomic and metabolomic data include the biological complexity of sex differences comprised of hormonal influences, genetic and epigenetic factors, and immune response [1]. Additional technical and methodological challenges contribute to the lack of conclusions in serum proteome and metabolome data, as both are highly complex, detecting both high- and low-abundance proteins and compounds, and there is difficulty in the attribution of differences to biological aspects compared to confounders including age and comorbidities [15]. The identification of biomarkers that are equally effective in women and men remains an understudied aspect of serum omics data. Given existing data supporting differences in outcomes (both progression and survival) in GBM in men vs. women, addressing these issues requires integrative, multidisciplinary approaches combining advanced proteomic and metabolomic technologies, robust bioinformatics, and the consideration of biological sex in study design and analysis. We wanted to define serum proteomic and metabolomic signals in a cohort of women and men with pathologically proven GBM using serum-derived proteomic and metabolomic data, hypothesizing that identified signals may be representative of both known signals associated with sex differences, which can provide validation of the methodology, and possible additional signals, which may indicate a relationship with the underlying malignancy, with possible downstream mechanistic insights. We repeated the same analysis in publicly available data (CPTAC and TCGA) and compared the results, exploring the intersectionality between serum vs. tissue in an effort to guide future efforts aimed at identifying GBM biomarkers with clinical applicability.

In this study, we proposed a hybrid feature selection method that combines LASSO (i.e., Least Absolute Shrinkage and Selection Operator) and mRMR (i.e., Minimum Redundancy Maximum Relevance). This approach leverages the advantages of sparse feature selection and reduced redundancy, effectively addressing the limitations of each method when applied individually. Through this integration, we sought to improve the reliability and interpretability of selected features, especially in the context of sex differences in glioblastoma. We also aimed to find the minimum number of selected features with the highest performance results in terms of accuracy rate.

The main contributions of this study, divided into technical and clinical aspects, are outlined below:

**Technical aspects**

- To the best of our knowledge, this is the first study that employs a combined feature selection and weighting methodology (i.e., GLIO-Select) employing female/male as a class label for classification tasks on different proteomic, metabolomic, and molecular datasets.
- To increase the scope and motivation of this study, we apply and compare our approach to the five different case studies on -omics and molecular datasets for classification.
- We adopted our previous MetaWise [16] methodology to analyze different types of problems and omics data.
- To address the effects of imbalanced class distribution in our datasets, we implemented stratified cross-validation, ensuring that each data fold retained the original class proportions.
- We utilized a rank-based feature weighting methodology to identify the feature names despite potential variations across cross-validation folds.
- We evaluated the effects of feature selection and weighting on six different machine learning models on proteomic, metabolomic, and molecular datasets to determine the optimal prediction model and minimal feature set for accurate classification.
- We visualized and interpreted selected features by using clustergram (i.e., dendrogram and heatmap) plots according to the dataset’s male/female class labels.

**Clinical aspects**

- We employed serum proteomic and metabolomic datasets applying feature selection operations to determine which serum signals distinguish samples obtained from females as compared to males. This is novel as serum proteomic and metabolomic profiles in GBM have not been described.
- To determine whether there are overlapping or linked signals between tissue and serum, we carried out the same analysis in CPTAC proteomic and metabolomic data.
- To link emerging signals and results derived using feature selection to transcriptomic data, we employed a TCGA glioma grading dataset we previously demonstrated as effective at glioma grading to identify mutations distinguishing tumor grade distribution between women and men.
- The present approach, coupled with interpretable dimensionality reduction (i.e., feature selection), enabled the identification of biomarkers with high accuracy to differentiate males from females in patients with GBM.
- Our methodology shows promising potential for future studies in several ways: (1) as a possible check for data accuracy in serum samples; (2) as a means of validating serum and tissue signals previously described using feature selection; (3) as a means of showcasing signals that have a distinct relationship to one sex vs. another, some of which represent novel signals; and (4) as a means of identifying signals that may help with further research into differential molecular and mechanistic aspects between females and males in GBM.

The remainder of this paper is structured as follows: Section 2 outlines the experimental setup, performance indicators, and computational outcomes in detail. Section 3 presents the results and discussion. Section 4 provides a detailed description of the dataset, the employed feature selection and weighting methodologies, and the supervised learning models used for classification. Finally, Section 5 summarizes the study’s findings and suggests potential directions for future research.

## 2. Results

In this section, we describe the experimental process and evaluation metrics, Then, we present our computational results based on rank-based feature selection and weighting in detail.

### 2.1. Experimental Process

To implement the proposed methods, we utilized Python’s scikit-learn library for machine learning algorithms and the mRMR [17] package for filter-based feature selection. We also utilized the MATLAB (version R2024b) environment for visualization purposes.

All experiments were carried out on a macOS Sequoia 15.2 MacBook Pro notebook with 16-core Apple M3 Max configurations and 128 GB LPDDR5 Memory. To achieve optimal results, we utilized six different classification models, including Support Vector Machines (SVMs), K-Nearest Neighbors (KNN), Logistic Regression (LR), Adaptive Boosting (AdaBoost), Random Forest (RF), and a voting-based ensemble learning model, which were used in both feature selection and classification stages. To ensure consistency across our previous studies [16,18] involving various data types, including molecular, proteomic, and metabolomic data, and given the numerous possible combinations of feature subsets, datasets, preprocessing methods, classification models, total or rank-ordered weights, and cross-validation methods, we have adopted the same parameter settings from Tasci et al. [16] and the same processing methods from our previous similar studies [16,18,19] for the feature selection and classification processes. In our study, we employed stratified five-fold cross-validation to evaluate the performance of our predictive models. In other words, the dataset is divided five-fold in a structured way (i.e., each fold maintains the same class distribution (male/female) as the original dataset). Each fold serves as a temporary hold-out set, while the remaining folds are used for model training. The mean accuracy rate value is obtained from the cross-validation process to calculate the performance results. This method was chosen for its ability to preserve class distributions across training and test sets, ensuring that male and female patient groups were proportionally represented in each fold. The cross-validation technique is also generally preferred and used over the hold-out set as it is considered more robust and better captures variations across different splits when the sample size is small. This was critical for minimizing bias and improving the robustness of our findings. Additionally, stratified cross-validation offered a computationally efficient approach to validate our results, making it particularly suitable given the scope of our study and the absence of hyperparameter optimization as a key objective. This methodology strikes a balance between computational efficiency and rigorous performance evaluation, thereby supporting the validity of our conclusions. The voting-based ensemble learning model consists of five prediction models, including SVM, KNN, LR, RF, and AdaBoost by applying the soft voting rule. We set the alpha parameter value as 0.01 for the LASSO feature selection (FS) method of the CPTAC [20] metabolomic and TCGA-UCI [21] GBM datasets due to the high correlation between features. To manage randomness and ensure consistent results on the datasets employed, we fixed the random state to 0 for all six machine-learning models.

### 2.2. Performance Metrics

To assess the performance of the hybrid filter and embedded feature selection techniques within the context of -omics data analysis, we specifically evaluated their classification accuracy. This metric directly reflects the success of the feature selection process in correctly classifying samples.

Classification accuracy (ACC) is determined by calculating the proportion of correctly classified samples within the entire dataset. This involves summing the number of true positive and true negative predictions and dividing this sum by the total number of samples, spanning both correct and incorrect classifications [22], as outlined in Equation (1).

(1) $$ACC=\frac{TP+TN}{TP+TN+FP+FN}$$

where TP, FN, TN, and FP denote the number of true positives, false negatives, true negatives, and false positives, respectively. This study aimed to find the best performance of different result sets according to the highest accuracy rate with the minimum number of selected features.

### 2.3. Computational Results

This subsection presents the effects of our feature selection and weighting approach on the performance of the learning models for five different datasets.

#### 2.3.1. The Effects of Feature Selection and Weighting Method on Classification Model Performance for Omics and Molecular Datasets in Patients with GBM

Using stratified five-fold cross-validation, we evaluated the performance of LASSO and mRMR-based feature selection methods using rank-based weighting schemes (with weights of 1 and 2). The computational results of these experiments, detailing the weight count (‘k’), are thoroughly tabulated in Table 1, Table 2, Table 3, Table 4 and Table 5. The changes in color, from red to green, in the tables denote the lowest (red) accuracy rate values to the highest accuracy rate values (green). Bold values show the best results in the related table.

- Proteomic Dataset Results

In this feature selection study for GBM patients, we obtained a 100% accuracy rate (ACC) with 17 selected proteomic features and the SVM classifier by assigning a rank value for LASSO of 2 and mRMR of 1 and using a weight value of 7 for the local preCRT-based proteomic dataset (see Table 1). A perfect classification accuracy score was yielded from a total of 7289 proteomic features for our serum-based GBM dataset with different numbers of selected features, models, and settings. We used the lowest number of selected features (i.e., largest dimensionality reduction) with the highest accuracy rate to provide efficient results. As can be observed in Table 1, LR also obtained a perfect accuracy rate by using 44 selected features and assigning weights of 1 to LASSO and 2 to mRMR. K-NN, RF, and voting-based ensemble learning models produced results close to the best and higher results than the AdaBoost model.

**Table 1:** The effect of the rank-based feature weighting and selection method on the accuracy rate (%) for the local preCRT-based proteomic dataset.

LASSO = 1 and mRMR = 2							
k	# of Features	SVM	LR	KNN	RF	AdaBoost	Voting
1	186	96.320	98.182	91.732	96.277	93.550	98.139
2	87	100.000	99.091	95.411	97.230	93.550	99.091
3	44	99.048	100.000	95.411	98.182	96.364	99.091
4	25	99.091	99.091	98.182	94.502	94.502	99.091
5	17	99.091	99.091	99.048	95.411	93.550	98.139
6	11	97.230	97.230	97.230	95.411	93.550	97.230
7	9	97.230	97.230	97.230	95.411	92.641	96.321
8	9	97.230	97.230	97.230	95.411	92.641	96.321
9	8	97.230	97.230	97.230	96.364	92.641	96.321
10	8	97.230	97.230	97.230	96.364	92.641	96.321
11	5	97.230	97.230	97.230	95.411	95.411	97.230
12	4	96.321	96.277	96.321	93.550	93.550	96.321
13	4	96.321	96.277	96.321	93.550	93.550	96.321
14	4	96.321	96.277	96.321	93.550	93.550	96.321
**LASSO = 2 and mRMR = 1**							
**k**	**# of Features**	**SVM**	**LR**	**KNN**	**RF**	**AdaBoost**	**Voting**
1	186	96.320	98.182	91.732	99.091	93.550	98.139
2	175	96.320	98.182	91.732	97.186	93.550	98.139
3	73	100.000	99.091	94.502	97.230	95.411	98.139
4	72	100.000	99.091	92.641	97.230	94.502	98.139
5	39	99.091	99.091	94.502	96.320	94.502	99.091
6	36	99.048	99.091	93.593	98.182	95.455	97.230
**7**	**17**	**100.000**	99.091	98.182	97.230	97.273	98.182
8	16	99.048	99.091	97.229	95.411	93.593	98.139
9	11	97.186	98.139	98.139	97.230	96.321	98.139
10	10	98.139	98.139	95.411	96.321	96.321	98.139
11	4	96.321	96.277	96.321	94.459	93.550	96.321
12	4	96.321	96.277	96.321	94.459	93.550	96.321
13	4	96.321	96.277	96.321	94.459	93.550	96.321
14	4	96.321	96.277	96.321	94.459	93.550	96.321

According to the CPTAC proteomic dataset feature selection results shown in Table 2, the best performance is obtained with 99% ACC, 5 selected proteomic features, and the SVM or KNN classifier by assigning a rank value of 2 to LASSO and 1 to mRMR and using a weight value of 6. As seen in the results in Table 2, all prediction models provided similar results. The AdaBoost model gave 97.000% ACC for all different weights and rank-based assignments. We propose that this status could be related to the effects of the features employed, data distribution, or model fitting. If we also use only two selected features, we can obtain 97% ACC for the CPTAC proteomic dataset.

**Table 2:** The effect of the rank-based feature weighting and selection method on the accuracy rate (%) for the CPTAC proteomic dataset.

LASSO = 1 and mRMR = 2							
k	# of Features	SVM	LR	KNN	RF	AdaBoost	Voting
1	68	98.000	99.000	96.947	99.000	97.000	98.000
2	51	99.000	99.000	98.000	99.000	97.000	99.000
3	14	99.000	98.000	99.000	99.000	97.000	98.000
4	11	99.000	98.000	99.000	98.000	97.000	98.000
5	7	98.000	98.000	98.000	97.947	97.000	98.000
6	7	98.000	98.000	98.000	97.947	97.000	98.000
7	6	97.000	97.000	97.000	98.000	97.000	98.000
8	6	97.000	97.000	97.000	98.000	97.000	98.000
9	4	97.000	97.000	97.000	98.000	97.000	98.000
10	4	97.000	97.000	97.000	98.000	97.000	98.000
11	2	97.000	97.000	98.000	98.000	97.000	98.000
12	2	97.000	97.000	98.000	98.000	97.000	98.000
13	2	97.000	97.000	98.000	98.000	97.000	98.000
14	2	97.000	97.000	98.000	98.000	97.000	98.000
**LASSO = 2 and mRMR = 1**							
**k**	**# of Features**	**SVM**	**LR**	**KNN**	**RF**	**AdaBoost**	**Voting**
1	68	98.000	99.000	96.947	99.000	97.000	98.000
2	36	98.000	99.000	97.000	99.000	97.000	99.000
3	16	98.000	99.000	98.000	99.000	97.000	99.000
4	15	98.000	99.000	97.000	98.000	97.000	99.000
5	8	99.000	99.000	99.000	98.000	97.000	98.000
**6**	**5**	**99.000**	98.000	**99.000**	96.947	97.000	98.000
7	2	97.000	97.000	98.000	98.000	97.000	98.000
8	2	97.000	97.000	98.000	98.000	97.000	98.000
9	2	97.000	97.000	98.000	98.000	97.000	98.000
10	2	97.000	97.000	98.000	98.000	97.000	98.000
11	2	97.000	97.000	98.000	98.000	97.000	98.000
12	2	97.000	97.000	98.000	98.000	97.000	98.000
13	2	97.000	97.000	98.000	98.000	97.000	98.000
14	2	97.000	97.000	98.000	98.000	97.000	98.000

- Metabolomic Dataset Results

We also evaluated our GLIO-Select methodology on two different metabolomic datasets. The effect of the rank-based feature weighting and selection method on the accuracy rate (%) for two datasets is presented in Table 3 and Table 4. The best performance is obtained with 91.6% ACC, 16 selected metabolomic features, and the LR classifier by assigning a rank value of 2 to LASSO and 1 to mRMR and using a weight value of 7 for the local level one confidence level preCRT-based metabolomic dataset.

**Table 3:** The effect of the rank-based feature weighting and selection method on the accuracy rate (%) for the local level one preCRT-based metabolomic dataset.

LASSO = 1 and mRMR = 2							
k	# of Features	SVM	LR	KNN	RF	AdaBoost	Voting
1	72	81.299	83.030	81.255	82.251	71.862	85.931
2	48	85.844	86.883	74.719	78.485	69.091	83.983
3	27	87.792	87.792	80.217	82.251	75.671	86.840
4	21	88.788	91.558	85.887	80.390	81.255	90.649
5	16	87.879	87.879	84.026	82.294	81.299	87.835
6	10	81.299	81.299	77.619	81.299	81.255	81.255
7	8	79.481	80.346	79.480	79.481	77.619	79.481
8	6	80.390	80.346	76.537	78.528	70.000	78.571
9	5	77.576	82.294	72.814	77.576	71.905	76.667
10	3	77.533	81.385	72.857	71.991	74.675	76.667
11	3	77.533	81.385	72.857	71.991	74.675	76.667
12	2	78.571	79.524	73.939	76.667	68.225	78.571
13	2	78.571	79.524	73.939	76.667	68.225	78.571
14	1	76.667	71.948	74.675	64.459	68.139	78.441
**LASSO = 2 and mRMR = 1**							
**k**	**# of Features**	**SVM**	**LR**	**KNN**	**RF**	**AdaBoost**	**Voting**
1	72	81.299	83.030	81.255	80.346	71.862	85.931
2	65	77.489	84.026	76.623	76.667	70.866	83.074
3	41	85.888	88.744	74.762	80.390	67.143	84.935
4	37	84.935	86.840	76.494	80.346	72.814	84.026
5	21	86.970	85.974	80.303	81.342	79.394	86.970
6	18	86.970	88.744	84.069	79.480	81.212	86.926
7	**16**	87.835	**91.558**	87.792	81.385	78.355	87.835
8	13	85.065	85.974	87.792	79.524	75.714	86.926
9	10	83.160	84.069	85.065	79.524	81.299	86.017
10	8	86.017	85.974	84.156	79.481	77.576	86.061
11	5	78.571	78.485	72.900	78.485	79.394	78.571
12	4	80.346	80.433	75.628	79.437	76.667	80.303
13	2	78.571	79.524	73.939	76.667	68.225	78.571
14	2	78.571	79.524	73.939	76.667	68.225	78.571

For the CPTAC metabolomic dataset, the best result is provided by 80% ACC and 13 selected metabolomic features by utilizing the LR or voting-based ensemble models, using the feature weight value of 10 and assigning a rank-based weight of 2 to LASSO and 1 to mRMR (see Table 4). For the proteomic and metabolomic datasets, the assignment of the rank-based weights of 2 to LASSO 2 and 1 to mRMR gave the highest accuracy rate for all combination sets of feature selection operations by employing different weights or prediction models.

**Table 4:** The effect of the rank-based feature weighting and selection method on the accuracy rate (%) for the CPTAC metabolomic dataset.

LASSO = 1 and mRMR = 2							
k	# of Features	SVM	LR	KNN	RF	AdaBoost	Voting
1	55	56.000	60.000	60.000	58.667	57.333	62.667
2	45	57.333	61.333	58.667	60.000	60.000	66.667
3	38	61.333	64.000	58.667	54.667	54.667	68.000
4	25	65.333	68.000	65.333	60.000	64.000	69.333
5	18	73.333	74.667	72.000	70.667	65.333	76.000
6	9	70.667	68.000	66.667	62.667	62.667	72.000
7	8	72.000	69.333	65.333	64.000	69.333	65.333
8	7	73.333	66.667	64.000	68.000	66.667	69.333
9	7	73.333	66.667	64.000	68.000	66.667	69.333
10	3	65.333	62.667	64.000	65.333	60.000	62.667
11	3	65.333	62.667	64.000	65.333	60.000	62.667
12	2	64.000	60.000	73.333	68.000	50.667	69.333
13	2	64.000	60.000	73.333	68.000	50.667	69.333
14	1	68.000	62.667	68.000	62.667	60.000	69.333
**LASSO = 2 and mRMR = 1**							
**k**	**# of Features**	**SVM**	**LR**	**KNN**	**RF**	**AdaBoost**	**Voting**
1	55	56.000	60.000	60.000	54.667	57.333	62.667
2	54	56.000	60.000	60.000	49.333	52.000	64.000
3	44	58.666	61.333	60.000	58.667	53.333	68.000
4	42	60.000	62.667	60.000	56.000	54.667	68.000
5	36	61.333	62.667	57.333	64.000	56.000	69.333
6	36	61.333	62.667	57.333	64.000	56.000	69.333
7	25	65.333	68.000	65.333	58.666	62.667	66.667
8	22	66.666	69.333	62.667	62.667	53.333	68.000
9	14	70.667	78.667	65.333	65.333	64.000	76.000
**10**	**13**	76.000	**80.000**	74.667	74.667	61.333	**80.000**
11	6	73.333	66.667	65.333	66.667	76.000	69.333
12	6	73.333	66.667	65.333	66.667	76.000	69.333
13	2	64.000	60.000	73.333	68.000	50.667	69.333
14	2	64.000	60.000	73.333	68.000	50.667	69.333

- Molecular Dataset Results

When we use the TCGA-GBM/UCI ML Repository molecular/mutation dataset for the male/female class label feature selection task, the best result is provided by 63.6% ACC, 3 selected features, a weight value of 11, and using SVM and RF models by assigning weights of 1 to LASSO and 2 to mRMR. The detailed results are illustrated in Table 5. This dataset shows which molecular/mutation genes are particularly discriminative for the current class label in GBM patient data. With the exception of KNN, all approaches provided similar results (Table 5) but were not able to separate the class label signals as well as the other datasets in this study. Overall, the three selected features resulted in 63.6% ACC.

**Table 5:** The effect of the rank-based feature weighting and selection method on the accuracy rate (%) for the TCGA-GBM/UCI ML Repository molecular dataset.

LASSO = 1 and mRMR = 2							
k	# of Features	SVM	LR	KNN	RF	AdaBoost	Voting
1	9	61.646	61.658	44.571	61.376	62.515	61.364
2	9	61.646	61.658	44.571	61.376	62.515	61.364
3	7	62.201	61.091	46.523	62.209	60.519	61.638
4	7	62.201	61.091	46.523	62.209	60.519	61.638
5	7	62.201	61.091	46.523	62.209	60.519	61.638
6	6	61.066	63.066	53.074	61.062	62.495	60.781
7	4	63.062	63.352	55.364	63.062	63.634	63.630
8	3	63.634	63.066	49.404	63.634	63.352	62.213
9	3	63.634	63.066	49.404	63.634	63.352	62.213
10	3	63.634	63.066	49.404	63.634	63.352	62.213
11	**3**	**63.634**	63.066	49.404	**63.634**	63.352	62.213
12	2	62.213	62.785	42.575	62.785	62.785	62.499
**LASSO = 2 and mRMR = 1**							
**k**	**# of Features**	**SVM**	**LR**	**KNN**	**RF**	**AdaBoost**	**Voting**
1	9	61.646	61.658	53.087	61.376	62.515	61.083
2	7	62.201	61.091	45.666	62.209	60.519	61.638
3	7	62.201	61.091	45.666	62.209	60.519	61.638
4	6	61.919	60.233	45.666	62.209	60.801	61.919
5	5	61.066	63.352	52.507	61.062	62.491	59.928
6	5	61.066	63.352	52.507	61.062	62.491	59.928
7	4	63.062	63.352	55.364	63.062	63.634	63.344
8	3	62.781	62.785	52.527	62.499	62.785	62.781
9	2	62.213	62.785	42.575	62.785	62.785	62.499
10	1	61.646	61.646	43.143	61.646	61.646	61.646
11	1	61.646	61.646	43.143	61.646	61.646	61.646
12	1	61.646	61.646	43.143	61.646	61.646	61.646

We give the selected feature names list for all datasets in Table S1 and present the mean performance of six prediction models on the datasets employed for the female/male dataset-based classification, with and without feature selection using five-fold stratified cross-validation in Supplementary Tables S2–S6. We assessed the performance of the different machine learning models using only LASSO FS or only mRMR FS methods for five GBM datasets. We observed that our feature selection and weighting methodology provided better results than using only one FS method.

#### 2.3.2. Our Best Results of the Utilized Datasets

We constructed a generic table to show our best results for the feature selection tasks in this study. Table 6 presents the best accuracy rates in percentage, the number of all features, and the number of selected features for the datasets used. The table shows that the largest dimensionality reduction is obtained with the CPTAC-GBM proteomic dataset by reducing the number of features from 8828 to 5. The best ACC is yielded by utilizing our local proteomic preCRT-based dataset as the perfect classification. The minimum selected number of features (i.e., 3) is obtained from the TCGA-UCI glioma grading dataset for GBM patients. The lowest dimensionality reduction rate (i.e., from 59 to 13) is obtained with the CPTAC-GBM metabolomic dataset.

### 2.4. Clinical Results

**Figure 1:**
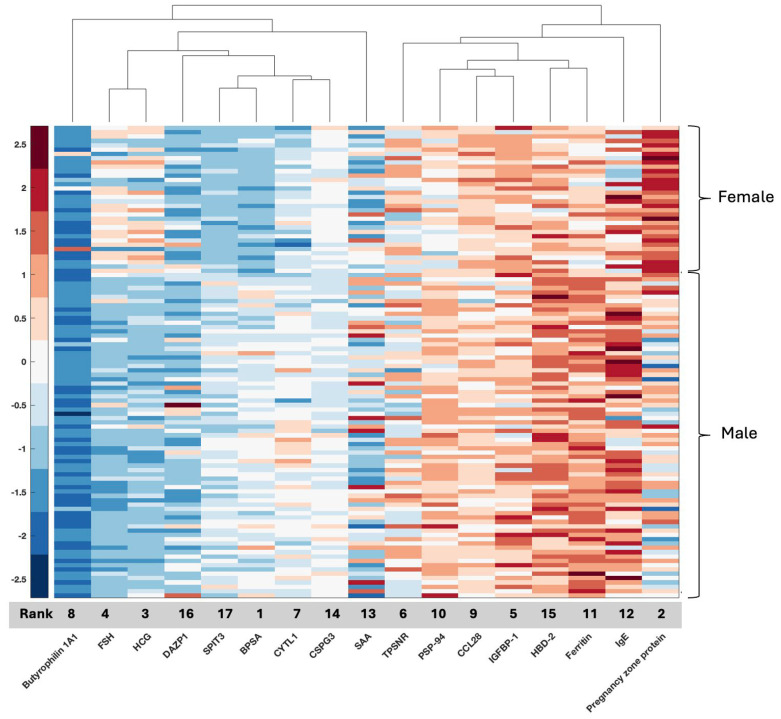
Clustergram for the selected features of the NCI NIH GBM proteomic dataset. Grey bar indicates the rank of the selected features from highest (1) to lowest (17) for the 17 selected features that were identified, resulting in a 100% accuracy rate.

**Figure 2:**
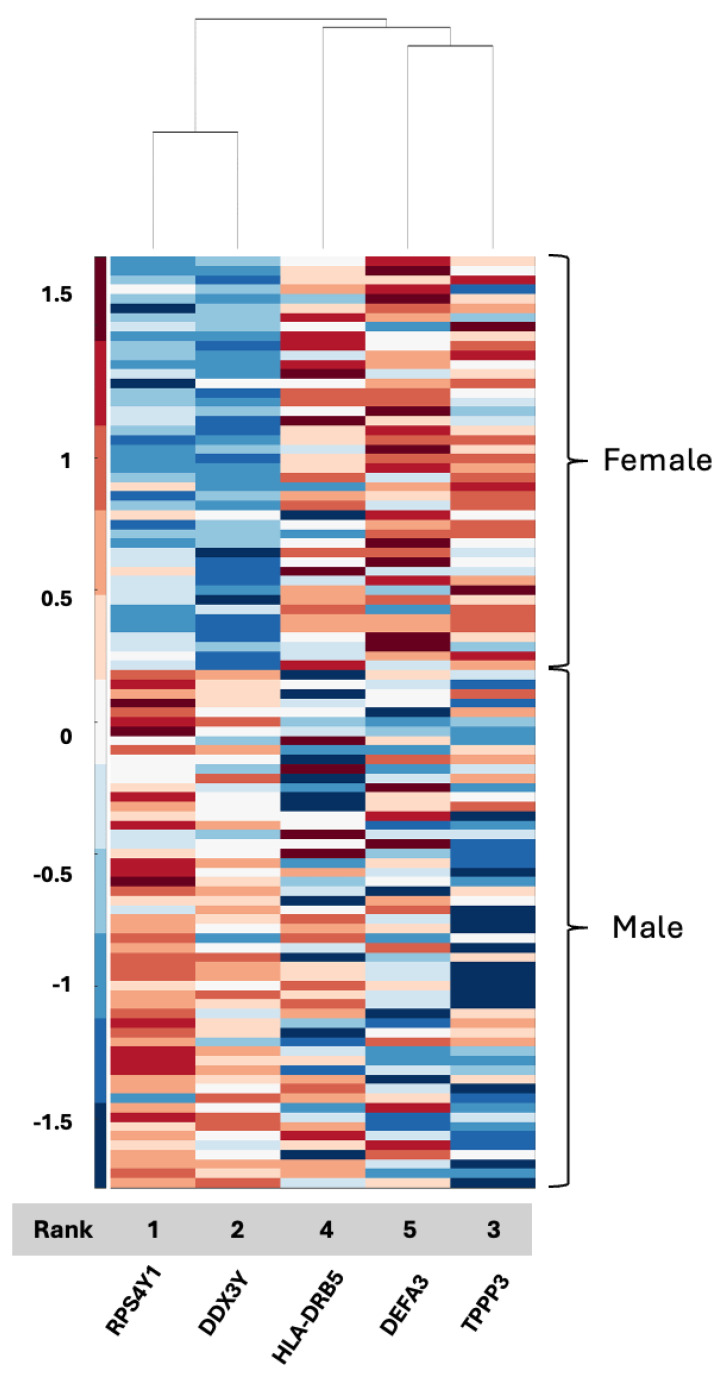
Clustergram for the selected features of the CPTAC proteomic dataset. Grey bar indicates the rank of the selected features from highest (1) to lowest (5) for the 5 selected features that were identified, resulting in a 99% accuracy rate.

**Figure 3:**
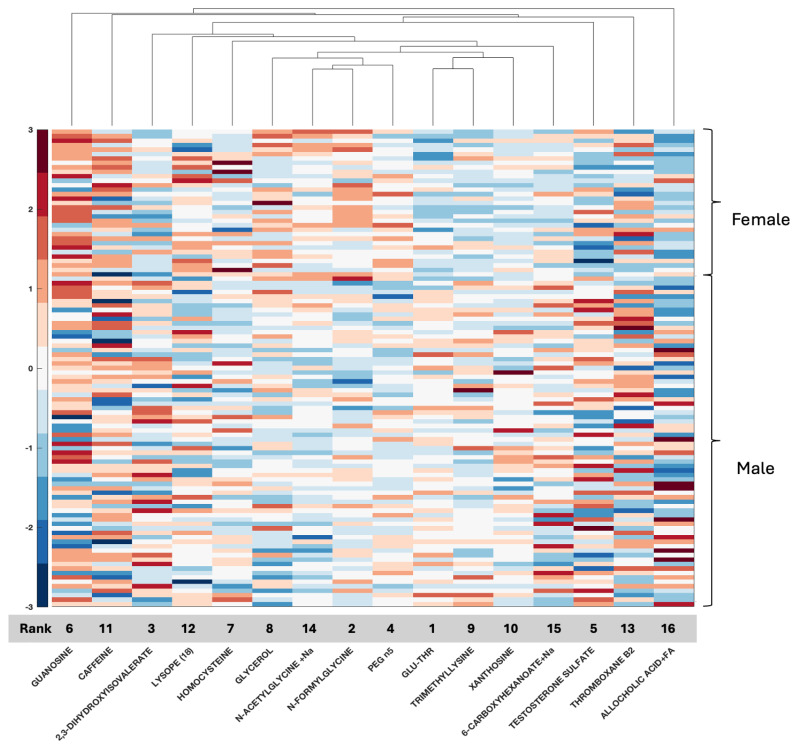
Clustergram for the selected features of the NCI NIH GBM serum metabolomic dataset. Grey bar indicates the rank of the selected features from highest (1) to lowest (16) for the 16 selected features that were identified, resulting in a 92% accuracy rate.

**Figure 4:**
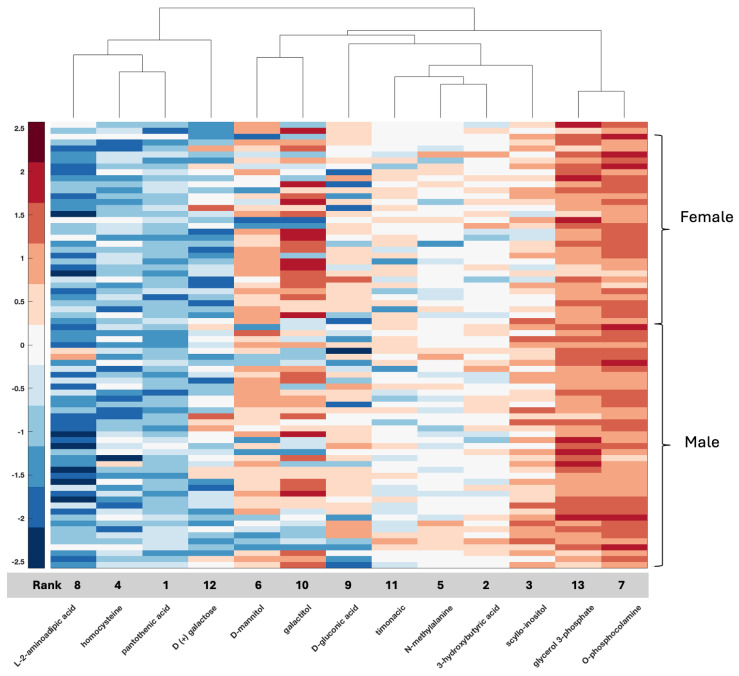
Clustergram for the selected features of the CPTAC tissue metabolomic dataset. Grey bar indicates the rank of the selected features from highest (1) to lowest (13) for the 13 selected features that were identified, resulting in an 80% accuracy rate.

**Figure 5:**
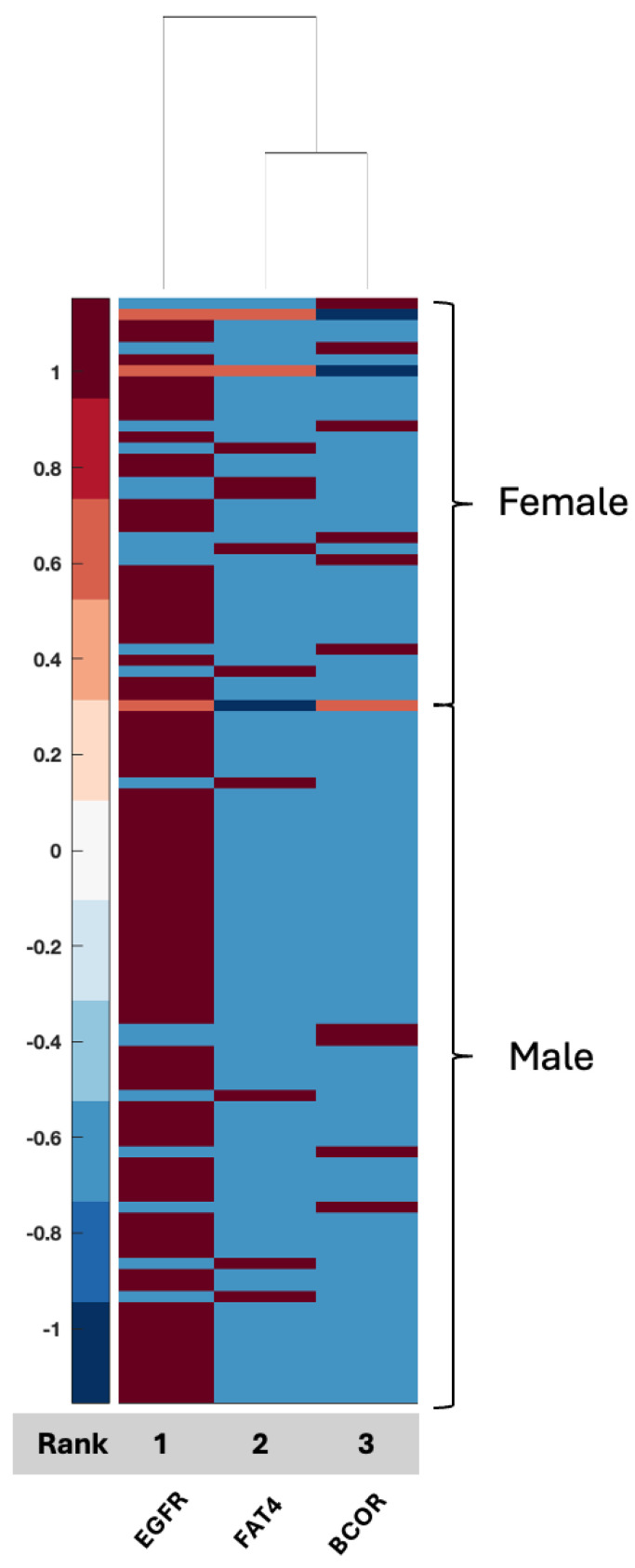
Clustergram for the selected features of the TCGA (UCI shared) dataset. Grey bar indicates the rank of the selected features from highest (1) to lowest (3) for the 3 selected features that were identified, resulting in a 64% accuracy rate.

We plotted clustergrams for each feature selection result in Figure 1, Figure 2, Figure 3, Figure 4 and Figure 5. Clustergrams are useful for grouping (i.e., hierarchical clustering) data based on similarity and visualizing those groupings. Clustergrams include a dendrogram and heatmap of the data. The colors and visual elements in a clustergram reveal key insights about the data structure. In clustergrams, colors usually represent the density or similarity of data points. Warm colors (reds and oranges) often indicate high density or similarity, while cool colors (blues and greens) suggest low density or similarity. The color scale helps illustrate how data points are distributed and their relative proximity. Clustergrams often consist of a dendrogram, a tree-like diagram that shows the sequence in which data points are grouped and how these groups are formed. By tracing the branches, we can identify which data points were grouped earlier and which were grouped later. The colors in the heatmap matrix represent the degree of similarity between two data points.

As can be shown in Figure 1, the most significant four features (96.3% ACC), namely, Benign Prostate-specific Antigen (BPSA), Pregnancy zone protein (PZP), Human Chorionic Gonadotropin (HCG), and Follicle-stimulating hormone (FSH), can have different values for males and females in our local proteomic dataset. It is observed that the BPSA heatmap value is around 0 in men and around −1 in women, and PZP in females has higher values than males. While the HCG and FSH heatmap values in females have positive values, it is observed that the values become negative for males. We also note the clustering of several markers. For example, two female-related sex hormones, FSH and HCG, exhibit high density in females and cluster together, while BPSA, a male marker, instead clusters with SPIT3 (Figure 1). HBD-2 and ferritin cluster together with lower values in females compared to males. CCL28 clusters with IGFBP-1, and then both cluster with PSP-94 (Figure 1).

As can be seen in Figure 2, the most discriminative two proteomic features (97% ACC) for the CPTAC dataset are RPS4Y1 and DDX3Y. Both of these features have “Y” annotating their known association with the male sex, so they cluster together and exhibit positive values in males and negative values emerge in female GBM patients. DEFA3 and TPPP3 cluster together with higher values in females and also cluster with HLA-DRB5, which exhibits a more ambiguous pattern (Figure 2). Both the local set and the CPTAC set result in nearly perfect accuracy with the exception of PZP and two components of the denfensin family (HBD-2, also known as DEFB4A, in serum and DEFA3 in CPTAC). The signals that emerged as the most significant features are different between the local set (serum) and CPTAC (tissue).

In the serum metabolomic data, the most significant two features (79.5% ACC) are obtained with GLU-THR and N-FORMYLGLYCINE for our local metabolomic dataset (Figure 3). While GLU-THR often has positive heatmap values in males with GBM, N-FORMYLGLYCINE has negative heatmap values in male GBM patients. The two most discriminative metabolomic features (60% ACC) are PANTOTHENIC ACID and 3-HYDROXYBUTYRIC ACID from the CPTAC metabolomic dataset (Figure 4). While PANTOTHENIC ACID heatmap data generally have a negative value in males or females, 3-HYDROXYBUTYRIC ACID has positive values or values around zero. The clustering of metabolic intermediates that share pathways (N-acetyl glycine and N-formylglycine, both elevated in females, and GLU-THR with TRYMETHYLYSINE, both decreased in females) also emerged (Figure 3). These metabolites are not present in the selected metabolic features in CPTAC. Two features were shared between the results in the two sets, HOMOCYSTEINE and PANTHOTHENIC ACID, although the latter did not make the list of top selected features that resulted in 92% accuracy in serum. In the CPTAC set, HOMOCYSTEINE and PANTHOTHENIC ACID cluster together, as do mannitol and galactitol, but differences between males and females are not as distinct in CPTAC tissue data, as evidenced by the 80% accuracy rate.

When employing the TCGA-UCI glioma grading dataset, the most discriminative and significant three features (63.6% ACC) are EGFR, FAT4, and BCOR. EGFR often has positive heatmap values for both men and women. However, FAT4 and BCOR often have negative heatmap values for both sexes (Figure 5). We can also say that heatmap values generally have lower or upper bound values as these mutation/molecular data contain only binary values. Additionally, as the highest ACC value is around 64%, it can be said that observing the differentiation in colors in males and females for the same molecules is difficult.

## 3. Discussion

While several studies have described sex differences in GBM employing transcriptomic or tissue-level proteomic data, noninvasive biospecimen-derived data and biological sex differences profiles in GBM serum proteomic and metabolomic data are not currently available [1,6,9,11,23]. This paucity of data limits the ability to account for the impact of biological sex when interpreting emerging biomarkers in serum while also limiting our mechanistic understanding of observed outcome differences. In this study, we identified several hundred serum features associated with the male and female class labels in proteomic and metabolomic datasets. Using the local serum-based dataset, 17 features (100% ACC) and 16 features (92% ACC) were identified for the proteomic and metabolomic datasets, respectively, demonstrating that the serum in our cohort is a robust biospecimen of analysis for the identification of proteins associated with sex differences. Using the CPTAC tissue-based dataset (8828 proteomic and 59 metabolomic features), 5 features (99% ACC) and 13 features (80% ACC) were identified for the proteomic and metabolomic datasets, respectively. The proteomic data serum (local data) or tissue (CPTAC) achieved the highest accuracy rates (100% and 99%, respectively), followed by serum metabolome (92% and 80%) for the local set and CPTAC, respectively (Table 6).

**Figure 6:**
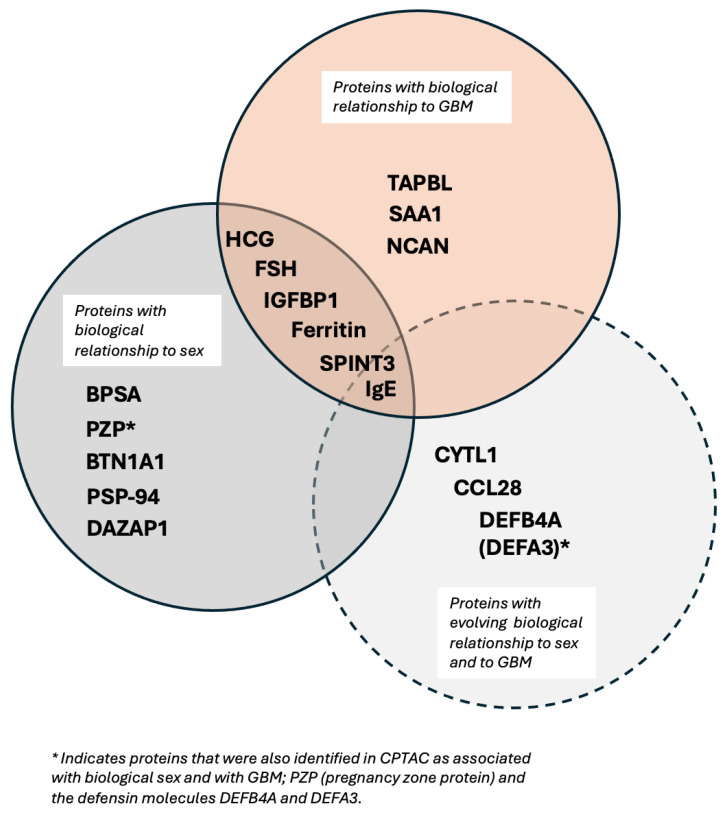
Proteins identified in serum have respective known relationships with biological sex and glioma, as well as novel signals with no direct or evolving relationships.

The current GLIO-Select method allowed for the selection of serum proteomic features that can differentiate a sample originating from a female GBM patient from a male GBM patient in serum with 100% accuracy for proteomic features and 92% accuracy for metabolomic features. The identified proteomic features offer both internal and methodological validation since several are already described as being associated with sex differences specifically in serum, including benign prostatic specific antigen (BPSA) [24], pregnancy zone protein (PZP) [25], PSP-94 [26], and DAZP1 [27]. Additional roles in glioma have also emerged for proteins known to have an association with one sex compared to the other, including HCG [28], FSH [29], PZP [25,30], and ferritin [31] (Table 7). However, while existing data support several of the identified features as being associated more with one sex than the other, for example, PZP with the female sex and PSA with the male sex, there are females with low PZP and males with PSA as low as that measured in most females. Thus, per the existing literature, no one serum protein or metabolite is 100% associated with either sex. For example, HCG may be measured in both men and women but may have physiological (pregnancy) or pathological (testicular cancer) implications. It should also be noted that just because certain serum proteins have a classical association with biological sex, such as HCG, they may have an additional role in GBM that is either unrecognized or subject to evolving data. HCG has been associated with GBM and may represent a possible biomarker [32], being implicated in glioma cells’ redox homeostasis. Equally so, there is an emerging relationship between sex hormones and androgen levels and tumor aggressiveness in GBM, which may be reflected in the identification of PSA, FSH, and PSP-94 [29,33]. Some proteins, such as IGFBP-1 (associated with stemness and invasion) [34] and BTN1A1 (novel immune checkpoint exclusive to PDL-1) [35] may have a relationship with biological sex [36], but this is evolving. In contrast, others are already associated with glioma (TPSNR (HLA1 antigen processing protein) [37], IgE [38], SAA1 (potential prognostic marker) [39], NCAN (also known as CSPG3, glycosylated chondroitin sulfate proteoglycan implicated in the tumor microenvironment) [40], and SPIT3 [41], and, in our analysis, are also associated with biological sex (Figure 6). Interestingly, SPIT3 clustered with BPSA in the serum, raising the hypothesis that there may be mechanistic features between the male sex and tumor behavior in males that may link to stemness and treatment resistance. DEFB4A (a component of the defensin family associated with several malignancies) [42] was identified in serum, while DEFA3 was identified in tissue, with their roles in innate immunity and malignancy evolving. These proteins present an opportunity to identify potential mechanistic relationships that drive glioma proliferation and response differences between the sexes since they are, in some form, present and associated with sex in both biospecimens.

PZP was also identified in CPTAC tissue proteome in this analysis; however, it was not one of the five features that directly contributed to ACC of 99%.

The CPTAC tissue proteome data achieved an accuracy rate of 99% resulting in five features: RPS4Y1, DDX3Y, HLA-DRB5, DEFA3, and TPPP3. Two of the features, RPS4Y1 and DDX3Y, are already Y chromosome-associated, and HLA-DRB5 and TPPP3 have been reported as being associated with GBM [43,44] with respect to prognosis and the epithelial–mesenchymal transition, respectively, albeit not in serum biospecimens as of yet. In contrast, DEFA3, a member of the defensin family (Supplemental Table S1), has not been directly implicated in GBM or associated with sex differences specifically. Defensins, however, have been connected to cancer [42,45], and their identification in both serum (DEFB4A) and tissue (DEFA3) in association with GBM and biological sex could support previous data suggesting a relationship between innate immunity as a critical aspect of GBM propagation [46] and its contribution to differential outcomes between men and women in GBM. Defensins captured in serum biospecimens thus merit additional research as a possible biomarker and a key to sex differences and differential responses to management. A connection between TPPP3, DEFA3, and HLA-DRB5, as evidenced by the clustering observed in the current analysis (Figure 3), had not yet been described, and if it presents as a distinguishing signaling axis between men and women with GBM, this merits additional investigation as it links the epithelial–mesenchymal transition to tumor grade and prognosis and the innate immune system, with a clear link to biological sex in the GBM tissue proteome. We also observed limited overlap between proteins identified in serum and proteins identified in tissue, which may indicate that one biospecimen cannot serve as a validation of signals for the other, although far more data are needed in GBM using patient samples that have both serum and tissue for analysis.

With respect to metabolomic data, the relationship between compounds in serum or tissue and biological sex is less well defined, although data are emerging. Compounds such as serum testosterone were identified in this study, again providing validation of the biospecimen of origin given clinical information with known clinical applicability for measurement in serum and supporting the accuracy of the feature selection method. Additional compounds such as GLU-THR (glutamine threonine), homocysteine, glycerol, xanthosine, and thromboxane B2 have also been associated with glioma and documented as differentially measured between men and women, while others such as allocholic acid are evolving (Supplemental Table S7) [1,4,47,48,49,50,51]. These compounds, as well as several others, relate to amino acid synthesis [52] and purine metabolism, as well as fatty acid metabolism [53]. Two metabolomic features shared between serum and tissue were homocysteine and pantothenic acid; however, overall, fewer compounds for analysis were present in CPTAC than in our dataset, with only 59 features to work with. Overall, both purine metabolism and fatty acid metabolism have been reported as highly significant in GBM, with emerging relationships to tumor resistance [53,54,55], and the current analysis supports that, indeed, several compounds may be captured in the serum and tumor tissue of patients with GBM, leading to potential mechanistic relationships for further research. Blood homocysteine levels are related to primary brain tumors [56] and homocysteine is associated with biological sex differences [47]. In the present study, homocysteine ranked fourth in the CPTAC tissue data (ACC 80%) and seventh in the local serum (ACC 92%). Pantothenic acid or vitamin B5 has been associated with GBM, and in a recent study, it was one of several metabolites differentially expressed between core and edge tumor specimens in association with MGMT status [57]. In the present study, it was ranked feature number 1 in tissue (CPTAC); however, it was ranked far lower in serum, not even reaching the 16 top identified features in serum. Since it emerged as a feature in both datasets, it warrants further study regarding its relationship with biological sex differences with respect to GBM.

**Figure 7:**
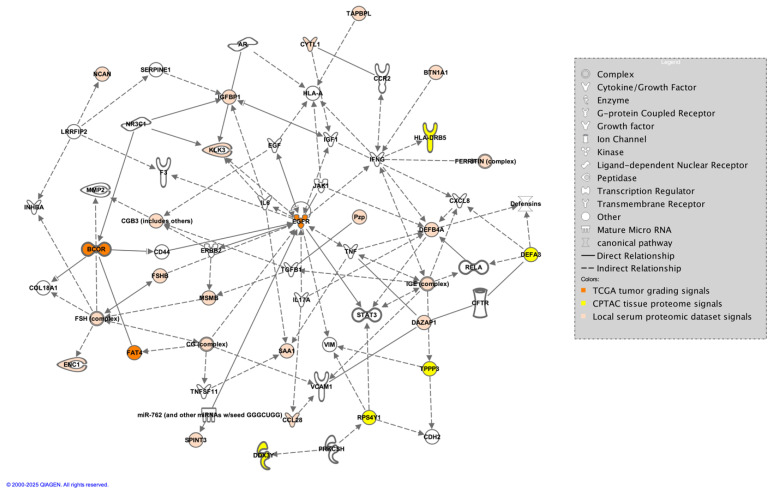
Direct and indirect connections between proteins identified in the TCGA tumor grading dataset (orange), the CPTAC tissue proteomic dataset (yellow), and the local serum dataset (tan) generated by the authors in IPA [62].

EGFR, FAT4, and BCOR were the three features associated with 64% ACC using the TCGA-UCI glioma grading set aimed at tumor grading [58]. The current analysis indicated that EGFR expression is differentially altered between men and women, with EGFR expression lower overall in women compared to men, which has been shown in other studies involving TCGA and other data [23]. FAT4 and BCOR, however, were overall higher in women as compared to men. Neither has been explored in their relationship with biological sex differences; however, FAT4 acts as a tumor-suppressor gene with typically lower expression in tumors [59], while BCOR (BCL6 corepressor gene) drives oncogenic transformation in neural cells [60]. BCOR has also been linked to glioma via BCOR-altered gliomas, a newly identified glioma with a potential response to fluorinated pyrimidines [61], indicating that glioma formation in women may occur via different pathways in women and men, resulting in differential tumor responses to chemotherapy. The relationship between the expression of these markers and sex differences is not well explored and the data are inconclusive. The current study illustrates potential relationships between well-known glioma drivers in males compared to females in GBM, some of which are better understood and studied; for example, the relationship between EGFR, BCOR, and FAT4 and connections to CD44 but with potential signaling via sex-related proteins such as HCG, as well as potential metabolic programming that may drive tumor behavior, e.g., via KLK3 (BPSA) and IGFBP1, which can connect to BCOR (Figure 7).

Our analyses can suggest that integrating LASSO with mRMR offers notable benefits compared to existing hybrid methods, such as enhanced classification performance or robustness. This highlights the effectiveness of our hybrid feature selection and weighting approach in managing high-dimensional data efficiently. Future studies will focus on refining and validating the proposed method using larger datasets and alternative hybrid techniques.

Our analysis focused on traditional machine learning methods, including SVM, KNN, Logistic Regression, AdaBoost, and Random Forest. Although modern techniques, such as deep neural networks, autoencoders, and transformer-based models, may offer benefits, these methods can introduce additional complexity and computational demands. Traditional approaches were chosen for their established utility in high-dimensional data with limited sample sizes, as well as their ease of interpretation. Future studies will explore integrating advanced techniques to compare their potential advantages with our current framework.

Some limitations of our study can be expressed as follows: The datasets used in our study do not include unaffected controls, which limits our ability to distinguish GBM-related features from those that may arise due to general variability unrelated to GBM. Future studies incorporating unaffected control data will be crucial for enhancing the specificity of identified features. While our feature selection analysis was aimed at identifying sex differences in GBM, it was conducted on combined datasets rather than separately for male and female groups. As another limitation of this study, we utilized two independent proteomic (or metabolomic) datasets to identify features associated with male/female GBM classification and feature selection. Although both datasets provided meaningful insights, the shared features between them were limited. This presented a challenge for implementing cross-dataset classification, which relies on consistent features across datasets for reliable evaluation. To address this, we concentrated on feature selection within each dataset, aiming to extract biologically relevant patterns unique to each one. Nonetheless, we recognize that cross-dataset classification could offer a more comprehensive assessment of the generalizability and robustness of our findings. Moving forward, future research can prioritize the inclusion of datasets with a higher degree of feature overlap or incorporate cross-dataset validation to reinforce our conclusions. Another limitation of this study is the lack of independent external validation, which is critical for assessing the generalizability of the model. The predictive model in this study was validated using stratified cross-validation as external validation was not possible due to the absence of datasets with the same specifications. This validation strategy ensures our results’ robustness within the available data’s constraints. Future work will aim to address this limitation by identifying or generating additional datasets to validate the findings externally.

Since a large number of classifier models, different datasets, five-fold stratified cross-validation technique, and different feature weighting values were tried and applied in this study, the implementation of the SHAP values in tree-based models or deep learning-based feature attribution methods would be associated with a significant increase in both time and processing power; it is, however, our intention to test these methods in future studies. While these advanced methods are recognized for both their capacity to capture complex nonlinear interactions and offer robust frameworks for feature selection, LASSO and mRMR were chosen for their well-established interpretability, computational efficiency, and suitability for high-dimensional omics datasets. Future work will address this limitation by comparing our approach with additional state-of-the-art techniques.

## 4. Materials and Methods

This section provides an overview and the key characteristics of our dataset employed. Subsequent subsections detail our methodological approach, including definitions, techniques, and prediction models utilized in this study.

### 4.1. Datasets

We employed a total of five proteomic, metabolomic, and molecular datasets from local or public data repositories to select the feature subset for the classification tasks in this study. The characteristics of the datasets employed are illustrated in Table 8.

The local proteomic preCRT-based dataset consists of NIH NCI ROB proteomic data of GBM patients with pre-chemoirradiation (CRT) status. This dataset is the format of log base two transformed regarding the preprocessing method and contains 109 cases with 74 males, 35 females, and 7289 proteomic features. The CPTAC (Clinical Proteomic Tumor Analysis Consortium)–GBM dataset is a nationwide initiative aimed at advancing the comprehension of cancer’s molecular underpinnings by leveraging extensive proteomic and genomic analyses, also known as proteogenomics [20]. The dataset includes different types of normalized GBM data, such as proteomic and metabolomic data. The local level one preCRT-based and normalized metabolomic dataset includes NIH NCI ROB metabolomic data from GBM patients who have not yet undergone chemoirradiation (CRT), adhering to confidence level one specification. The TCGA-UCI glioma grading dataset [21] for GBM patients includes only GBM patient data with 20 significant molecular features for 352 patients. These features can be not_mutated (i.e., 0) or mutated (i.e., 1) depending on their status.

**Table 8:** Proteomic, metabolomic, and molecular feature selection datasets utilized for the classification tasks.

Dataset	Preprocessing	# of Cases	# of Males	# of Females	# of Features
Local Proteomic PreCRT-based Dataset	Log Base 2 Transformed	109	74 (68%)	35 (32%)	7289
CPTAC-GBM Proteomic Dataset	Normalized	99	55 (56%)	44 (44%)	8828
CPTAC-GBM Metabolomic Dataset	Normalized	75	41 (55%)	34 (45%)	59
Local Level One PreCRT-based Metabolomic Dataset	Normalized	107	73 (68%)	34 (32%)	318
TCGA-UCI Glioma Grading Dataset for GBM	No Preprocessing	352	217 (62%)	135 (38%)	20

### 4.2. Methodology

This section provides a general overview of our employed feature selection and weighting architecture, consisting of a brief description of underpinning methodologies.

#### 4.2.1. Proposed Scheme

**Figure 8:**
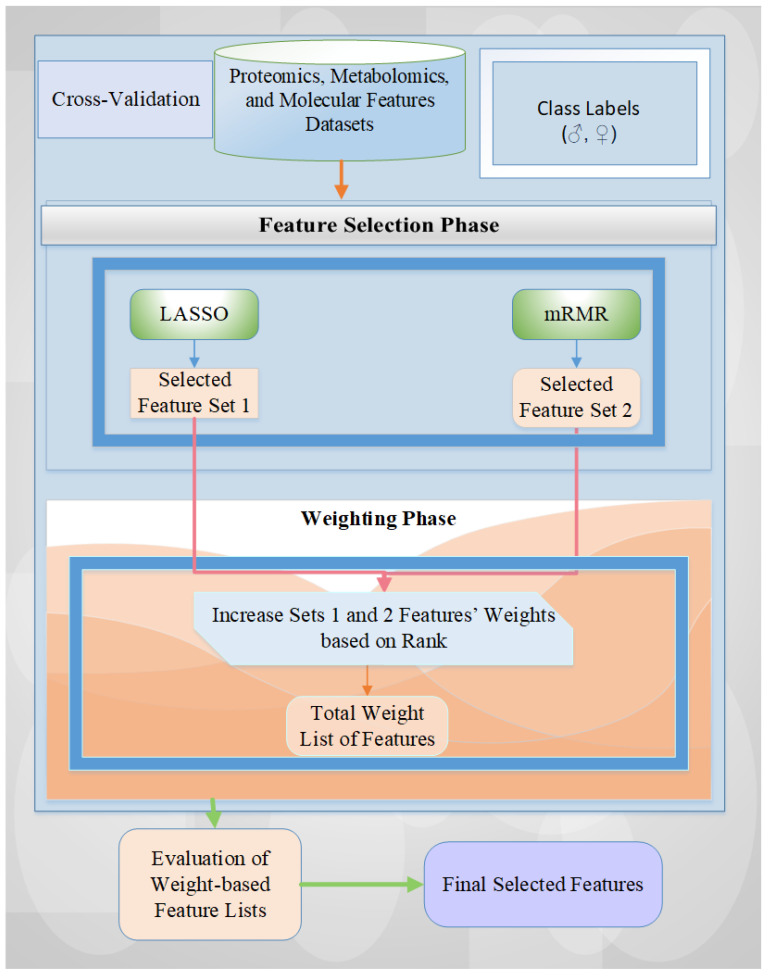
General overview of the utilized architecture for feature selection tasks.

In this study, we adopted our previous methodology, MetaWise [16], with the same settings for feature selection tasks on five different GBM datasets that captured male and female as class labels for analysis. Our utilized scheme consists of two phases: (a) feature selection (FS) and (b) feature weighting (FW) [18,58,63]. These stages use the fusion of the two feature selection methods: Least Absolute Shrinkage and Selection Operator (LASSO) and Minimum Redundancy Maximum Relevance (mRMR). The detailed schematic diagram is illustrated in Figure 8.

First, all features are entered into the feature selection (FS) model using a cross-validation technique. For each fold, the feature sets selected by the two FS methods are saved into variables, and the selected feature counts are summed according to the weights provided by the rank-based approach (i.e., 2 or 1 for two FS methods). Then, the weight-based feature list is evaluated by using all weight values (i.e., features with higher weight values represent high importance levels). In the last phase, the final feature list is derived by examining all weight values and identifying those with the highest accuracy rate across six machine learning models: Support Vector Machine (SVM), K-Nearest Neighbors (KNN), Logistic Regression (LR), Random Forest (RF), Adaptive Boosting (AdaBoost), and voting-based ensemble learning model. The combined strategy of using LASSO and mRMR FS methods provides dimensionality reduction and contributes to decreasing feature redundancy with high-performance results by balancing feature relevance between the target and them. For an in-depth explanation of this methodology, please refer to Tasci et al. [16].

#### 4.2.2. Feature Selection Methods

Feature selection is the process of selecting a subset of relevant and discriminative features for use in learning model construction. This step is crucial to improve the prediction model’s performance by eliminating redundant or irrelevant data, which can enhance the accuracy, speed, and interpretability of the model [19,64]. Feature selection methods can be categorized into three classes based on the evaluation criteria: filter, wrapper, and embedded methods [64]. Filter feature selection methods assess the relevance of features by examining metrics such as their statistical relationships with the target variable. They are independent of any machine learning algorithm and include techniques like correlation coefficients (e.g., mRMR). They are typically fast and computationally efficient but might ignore feature interactions. Wrapper feature selection methods evaluate the feature subset by training a model on different combinations of features and selecting the subset that yields the best performance. These methods are computationally expensive because they involve training multiple learning models. Embedded feature selection methods perform feature selection during the model training process (e.g., LASSO). Common methods consist of LASSO (Least Absolute Shrinkage and Selection Operator), which shrinks coefficients of less important features to zero, and mRMR (Minimum Redundancy Maximum Relevance), which selects features that have minimal redundancy among the features and maximum relevance to the target variable. These methods help in identifying the most significant feature subset that contributes to the predictive power of the learning model.

#### 4.2.3. Feature Weighting Methods

To determine the relative importance of selected features in identifying patterns, weights were assigned based on their contribution to the classification operation in terms of their accuracy rate. A rank-based feature weighting scheme was applied for the LASSO and mRMR FS methods in this study. Their performance (i.e., accuracy rate) on each data fold was used to rank them. The top-performing method was given a weight of 2, while the other was assigned a weight of 1 for each fold. This process was repeated for all possible rank combinations to optimize feature weights for all the datasets [16]. In the final stage, all selected feature weights are summed and assessed to find the best-performing feature subset with the smallest number of features.

### 4.3. Classification

The classification stage involves training a machine learning model to categorize data into predefined classes or labels. Once the learning model is trained, the model can predict the class of new and unseen data based on the learned patterns. In our previous works [16,18], we provided a comprehensive overview of popular classification algorithms such as KNN, SVM, LR, AdaBoost, RF, and voting-based ensemble learning models. We employed a soft voting-based approach that predicts the class label based on the maximum of the sums of the predicted probabilities for five classification models [65].

## 5. Conclusions

This study (i.e., GLIO-Select) carries a fusion of the filter and embedded feature selection and weighting methodologies for feature selection tasks on five different GBM high-dimensional omics or molecular datasets that capture female and male as class labels. Six different machine-learning model performances are assessed regarding the accuracy rate with the different number of weights or selected features. Serum and tissue-based dataset results are also analyzed for feature selection operations. The critical importance level of the feature selection process regarding model performance improvement and reducing the computational complexity is also observed in detail for the dimensionality reduction operations. Overall, several signals emerged in the current analysis, which are known to be associated with glioma or GBM but were previously not known to be associated with biological sex, with several signals present in non-invasively derived biospecimen (serum). These findings require further validation and merit further scrutiny as potential therapies grounded in several critical signaling and metabolic pathways; notably, amino acid and fatty acid metabolism may have differential expression between men and women and result in different therapeutic responses. The ability to detect serum proteins and metabolites that are different between women and men provides encouragement that noninvasive biospecimen collection may provide biomarkers that can effectively eventually lead to more personalized management in GBM. Several novel signals that were previously not linked to either biological sex or glioma also emerged in the analysis and may relate to mechanistic insights for differential tumor responses between the sexes, especially those that link mechanistically to both tissue and genomic data.

By focusing on the most relevant and discriminative features, researchers can build more accurate and efficient models and aid in early diagnosis, prognosis, and personalized treatment of GBM. The feature selection process can also mitigate the curse of dimensionality and improve the generalizability of learning models. Ongoing advancements and research in this area are likely to significantly enhance patient outcomes and make substantial contributions to the broader field of cancer research regarding biomarker discovery or personalized treatments.

## Figures and Tables

**Table 6:** Our best results of the employed datasets for GBM (all identified features are in Supplementary Table S1).

Dataset	# of Features	Accuracy Rate (%)	Number of Identified Features
Local Proteomic PreCRT-based Dataset	7289	100.000	17
CPTAC-GBM Proteomic Dataset	8828	99.000	5
CPTAC-GBM Metabolomic Dataset	59	80.000	13
Local Level One PreCRT-based Metabolomic Dataset	318	91.558	16
TCGA-UCI Glioma Grading Dataset for GBM	20	63.634	3

**Table 7:** The 16 proteomic features identified in the NCI NIH GBM serum proteomic dataset. Green fill indicates known association with biological sex based on existing literature and use in the clinic and/or association with glioma or, more specifically in some cases, glioblastoma (GBM).

Target	Entrez Gene Symbol	Target Full Name	Documented Association with Biological Sex	Documented Association with Glioma
BPSA	KLK3	Benign Prostate specific Antigen	yes [24]	no
PZP	PZP	Pregnancy zone protein	yes [25]	no
HCG	CGA|CGB3|CGB7	Human Chorionic Gonadotropin	yes [28]	yes [32]
FSH	CGA|FSHB	Follicle stimulating hormone	yes	yes [29]
IGFBP-1	IGFBP1	Insulin-like growth factor-binding protein 1	possible [36]	yes [34]
TPSNR	TAPBPL	Tapasin-related protein	no	yes [37]
CYTL1	CYTL1	Cytokine-like protein 1	no	no
BTN1A1	BTN1A1	Butyrophilin subfamily 1 member A1	possible [35]	no
CCL28	CCL28	C-C motif chemokine 28	no	no
PSP-94	MSMB	Beta-microseminoprotein	yes [29,33]	no
Ferritin	FTH1|FTL	Ferritin	yes [1]	yes [31]
IgE	IGHE	Immunoglobulin E	possible	yes [38]
SAA1	SAA1	Serum amyloid A-1 protein	no	yes [39]
CSPG3	NCAN	Neurocan core protein	no	yes [40]
HBD-2	DEFB4A	Beta-defensin 4A	no	no
DAZP1	DAZAP1	DAZ-associated protein 1	yes [27]	no
SPIT3	SPINT3	Kunitz-type protease inhibitor 3	no	yes [41]

## Data Availability

We have attached the organized CPTAC proteomic, metabolomic, and TCGA-UCI Glioma Grading for GBM datasets. De-identified data, including clinical data associated with our local omics dataset, will be shared once analyses for outcomes are complete.

## Supplementary Materials

The following supporting information can be downloaded at: https://www.mdpi.com/article/10.3390/ijms26094339/s1.

## Abbreviations

ACC	Accuracy
AdaBoost	Adaptive Boosting
CPTAC	Clinical Proteomic Tumor Analysis Consortium
CRT	Chemoirradiation
FS	Feature Selection
FW	Feature Weighting
GBM	Glioblastoma Multiforme
KNN	K Nearest Neighbors
IPA	Ingenuity Pathway Analysis
LASSO	Least Absolute Shrinkage and Selection Operator
LR	Logistic Regression
MRMR	Minimum Redundancy—Maximum Relevance
NCI	National Cancer Institute
NIDAP	NIH Integrated Data Analysis Platform
NIH	National Institutes of Health
RF	Random Forest
ROB	Radiation Oncology Branch
RT	Radiation Therapy
SECIM	Southeast Center for Integrated Metabolomics
SVM	Support Vector Machine
TMZ	Temozolomide

## References

[1] Carrano A., Juarez J.J., Incontri D., Ibarra A., Guerrero Cazares H. (2021). Sex-Specific Differences in Glioblastoma. Cells.

[2] Massey S.C., Whitmire P., Doyle T.E., Ippolito J.E., Mrugala M.M., Hu L.S., Canoll P., Anderson A.R.A., Wilson M.A., Fitzpatrick S.M. (2021). Sex differences in health and disease: A review of biological sex differences relevant to cancer with a spotlight on glioma. Cancer Lett..

[3] Ippolito J.E., Yim A.K., Luo J., Chinnaiyan P., Rubin J.B. (2017). Sexual dimorphism in glioma glycolysis underlies sex differences in survival. JCI Insight.

[4] Sponagel J., Jones J.K., Frankfater C., Zhang S., Tung O., Cho K., Tinkum K.L., Gass H., Nunez E., Spitz D.R. (2022). Sex differences in brain tumor glutamine metabolism reveal sex-specific vulnerabilities to treatment. Med.

[5] Yang W., Warrington N.M., Taylor S.J., Whitmire P., Carrasco E., Singleton K.W., Wu N., Lathia J.D., Berens M.E., Kim A.H. (2019). Sex differences in GBM revealed by analysis of patient imaging, transcriptome, and survival data. Sci. Transl. Med..

[6] Jovanovich N., Habib A., Chilukuri A., Hameed N.U.F., Deng H., Shanahan R., Head J.R., Zinn P.O. (2023). Sex-specific molecular differences in glioblastoma: Assessing the clinical significance of genetic variants. Front. Oncol..

[7] Lee J., Kay K., Troike K., Ahluwalia M.S., Lathia J.D. (2022). Sex Differences in Glioblastoma Immunotherapy Response. Neuromolecular Med..

[8] Lee J., Troike K., Fodor R., Lathia J.D. (2022). Unexplored Functions of Sex Hormones in Glioblastoma Cancer Stem Cells. Endocrinology.

[9] Shireman J.M., Ammanuel S., Eickhoff J.C., Dey M. (2022). Sexual dimorphism of the immune system predicts clinical outcomes in glioblastoma immunotherapy: A systematic review and meta-analysis. Neuro-Oncol. Adv..

[10] Tavelin B., Malmstrom A. (2022). Sex Differences in Glioblastoma-Findings from the Swedish National Quality Registry for Primary Brain Tumors between 1999-2018. J. Clin. Med..

[11] Garcia C.A., Bhargav A.G., Brooks M., Suarez-Meade P., Mondal S.K., Zarco N., ReFaey K., Jentoft M., Middlebrooks E.H., Snuderl M. (2021). Functional Characterization of Brain Tumor-Initiating Cells and Establishment of GBM Preclinical Models that Incorporate Heterogeneity, Therapy, and Sex Differences. Mol. Cancer Ther..

[12] Zhou J., Guo H., Liu L., Hao S., Guo Z., Zhang F., Gao Y., Wang Z., Zhang W. (2021). Construction of co-expression modules related to survival by WGCNA and identification of potential prognostic biomarkers in glioblastoma. J. Cell Mol. Med..

[13] Linhares P., Carvalho B., Vaz R., Costa B.M. (2020). Glioblastoma: Is There Any Blood Biomarker with True Clinical Relevance?. Int. J. Mol. Sci..

[14] Diaz Rosario M., Kaur H., Tasci E., Shankavaram U., Sproull M., Zhuge Y., Camphausen K., Krauze A. (2022). The Next Frontier in Health Disparities—A Closer Look at Exploring Sex Differences in Glioma Data and Omics Analysis, from Bench to Bedside and Back. Biomolecules.

[15] Krauze A.V., Sierk M., Nguyen T., Chen Q., Yan C., Hu Y., Jiang W., Tasci E., Zgela T.C., Sproull M. (2023). Glioblastoma survival is associated with distinct proteomic alteration signatures post chemoirradiation in a large-scale proteomic panel. Front. Oncol..

[16] Tasci E., Popa M., Zhuge Y., Chappidi S., Zhang L., Zgela T.C., Sproull M., Mackey M., Kates H.R., Garrett T.J. (2024). MetaWise: Combined Feature Selection and Weighting Method to Link the Serum Metabolome to Treatment Response and Survival in Glioblastoma. Int. J. Mol. Sci..

[17] mRMR Feature Selection. https://github.com/smazzanti/mrmr.

[18] Tasci E., Shah Y., Jagasia S., Zhuge Y., Shephard J., Johnson M.O., Elemento O., Joyce T., Chappidi S., Cooley Zgela T. (2024). MGMT ProFWise: Unlocking a New Application for Combined Feature Selection and the Rank-Based Weighting Method to Link MGMT Methylation Status to Serum Protein Expression in Patients with Glioblastoma. Int. J. Mol. Sci..

[19] Tasci E., Zhuge Y., Kaur H., Camphausen K., Krauze A.V. (2022). Hierarchical Voting-Based Feature Selection and Ensemble Learning Model Scheme for Glioma Grading with Clinical and Molecular Characteristics. Int. J. Mol. Sci..

[20] Wang L.-B., Karpova A., Gritsenko M.A., Kyle J.E., Cao S., Li Y., Rykunov D., Colaprico A., Rothstein J.H., Hong R. (2021). Proteogenomic and metabolomic characterization of human glioblastoma. Cancer Cell.

[21] Tasci E., Zhuge Y., Camphausen K., Krauze A.V. Glioma Grading Clinical and Mutation Features Dataset. 2022.

[22] Fawcett T. (2006). An introduction to ROC analysis. Pattern Recognit. Lett..

[23] Jang B., Yoon D., Lee J.Y., Kim J., Hong J., Koo H., Sa J.K. (2024). Integrative multi-omics characterization reveals sex differences in glioblastoma. Biol. Sex Differ..

[24] Linton H.J., Marks L.S., Millar L.S., Knott C.L., Rittenhouse H.G., Mikolajczyk S.D. (2003). Benign prostate-specific antigen (BPSA) in serum is increased in benign prostate disease. Clin. Chem..

[25] Yang J., Fang W., Wu W., Tian Z., Gao R., Yu L., Chen D., Weng X., Zhu S., Yang C. (2021). A Novel Diagnostic Biomarker, PZP, for Detecting Colorectal Cancer in Type 2 Diabetes Mellitus Patients Identified by Serum-Based Mass Spectrometry. Front. Mol. Biosci..

[26] Luebke A.M., Attarchi-Tehrani A., Meiners J., Hube-Magg C., Lang D.S., Kluth M., Tsourlakis M.C., Minner S., Simon R., Sauter G. (2019). Loss of PSP94 expression is associated with early PSA recurrence and deteriorates outcome of PTEN deleted prostate cancers. Cancer Biol. Med..

[27] Tsui S., Dai T., Roettger S., Schempp W., Salido E.C., Yen P.H. (2000). Identification of Two Novel Proteins That Interact with Germ-Cell-Specific RNA-Binding Proteins DAZ and DAZL1. Genomics.

[28] Kros J.M., Mustafa D.M., Dekker L.J., Sillevis Smitt P.A., Luider T.M., Zheng P.P. (2015). Circulating glioma biomarkers. Neuro-Oncol..

[29] Fariña-Jerónimo H., Martín-Ramírez R., González-Fernández R., Medina L., de Vera A., Martín-Vasallo P., Plata-Bello J. (2024). Androgen deficiency is associated with a better prognosis in glioblastoma. Eur. J. Med. Res..

[30] Huang J., Xu Y., Chen Y., Shen J., Qiu Y., Li X., Chen X., Ma S. (2024). Revisiting the role of pregnancy zone protein (PZP) as a cancer biomarker in the immunotherapy era. J. Transl. Med..

[31] Jaksch-Bogensperger H., Spiegl-Kreinecker S., Arosio P., Eckl P., Golaszewski S., Ebner Y., Al-Schameri R., Strasser P., Weis S., Bresgen N. (2020). Ferritin in glioblastoma. Br. J. Cancer.

[32] Ahmad F., Ghosh S., Sinha S., Joshi S.D., Mehta V.S., Sen E. (2015). TGF-β-induced hCG-β regulates redox homeostasis in glioma cells. Mol. Cell. Biochem..

[33] Mondragón J.A., Serrano Y., Torres A., Orozco M., Segovia J., Manjarrez G., Romano M.C. (2021). Glioblastoma cells express crucial enzymes involved in androgen synthesis: 3β-hydroxysteroid dehydrogenase, 17-20α-hydroxylase, 17β-hydroxysteroid dehydrogenase and 5α-reductase. Endocrinol. Diabetes Metab..

[34] Liu Z., Ji H., Fu W., Ma S., Zhao H., Wang F., Dong J., Yan X., Zhang J., Wang N. (2022). IGFBPs were associated with stemness, inflammation, extracellular matrix remodeling and poor prognosis of low-grade glioma. Front. Endocrinol..

[35] Kim Y.S., Lee S.H., Park A.H., Wu C., Hong B.K., Jung H., Lin S.H., Yoo S.S. (2024). BTN1A1 is a novel immune checkpoint mutually exclusive to PD-L1. J. Immunother. Cancer.

[36] Undén A.L., Elofsson S., Brismar K. (2005). Gender differences in the relation of insulin-like growth factor binding protein-1 to cardiovascular risk factors: A population-based study. Clin. Endocrinol..

[37] Thuring C., Follin E., Geironson L., Freyhult E., Junghans V., Harndahl M., Buus S., Paulsson K.M. (2015). HLA class I is most tightly linked to levels of tapasin compared with other antigen-processing proteins in glioblastoma. Br. J. Cancer.

[38] Guerra G., Nakase T., Kachuri L., McCoy L., Hansen H.M., Rice T., Wiemels J.L., Wiencke J.K., Molinaro A.M., Wrensch M. (2024). Association of immunoglobulin E levels with glioma risk and survival. J. Natl. Cancer Inst..

[39] Cao K., Jiang X., Wang B., Ni Z., Chen Y. (2022). SAA1 Expression as a Potential Prognostic Marker of the Tumor Microenvironment in Glioblastoma. Front. Neurol..

[40] Silver D.J., Siebzehnrubl F.A., Schildts M.J., Yachnis A.T., Smith G.M., Smith A.A., Scheffler B., Reynolds B.A., Silver J., Steindler D.A. (2013). Chondroitin sulfate proteoglycans potently inhibit invasion and serve as a central organizer of the brain tumor microenvironment. J. Neurosci..

[41] Pang L., Dunterman M., Guo S., Khan F., Liu Y., Taefi E., Bahrami A., Geula C., Hsu W.H., Horbinski C. (2023). Kunitz-type protease inhibitor TFPI2 remodels stemness and immunosuppressive tumor microenvironment in glioblastoma. Nat. Immunol..

[42] Adyns L., Proost P., Struyf S. (2023). Role of Defensins in Tumor Biology. Int. J. Mol. Sci..

[43] Fan X., Liang J., Wu Z., Shan X., Qiao H., Jiang T. (2017). Expression of HLA-DR genes in gliomas: Correlation with clinicopathological features and prognosis. Chin. Neurosurg. J..

[44] Xu X., Hou Y., Long N., Jiang L., Yan Z., Xu Y., Lv Y., Xiang X., Yang H., Liu J. (2023). TPPP3 promote epithelial-mesenchymal transition via Snail1 in glioblastoma. Sci. Rep..

[45] Ghosh S.K., McCormick T.S., Weinberg A. (2019). Human Beta Defensins and Cancer: Contradictions and Common Ground. Front. Oncol..

[46] Gillard A.G., Shin D.H., Hampton L.A., Lopez-Rivas A., Parthasarathy A., Fueyo J., Gomez-Manzano C. (2024). Targeting Innate Immunity in Glioma Therapy. Int. J. Mol. Sci..

[47] Cohen E., Margalit I., Shochat T., Goldberg E., Krause I. (2019). Gender differences in homocysteine concentrations, a population-based cross-sectional study. Nutr. Metab. Cardiovasc. Dis..

[48] Bolognesi A., Bortolotti M., Battelli M.G., Polito L. (2024). Gender Influence on XOR Activities and Related Pathologies: A Narrative Review. Antioxidants.

[49] Campanella R., Guarnaccia L., Cordiglieri C., Trombetta E., Caroli M., Carrabba G., La Verde N., Rampini P., Gaudino C., Costa A. (2020). Tumor-Educated Platelets and Angiogenesis in Glioblastoma: Another Brick in the Wall for Novel Prognostic and Targetable Biomarkers, Changing the Vision from a Localized Tumor to a Systemic Pathology. Cells.

[50] Osuna-Prieto F.J., Rubio-Lopez J., Di X., Yang W., Kohler I., Rensen P.C.N., Ruiz J.R., Martinez-Tellez B. (2022). Plasma Levels of Bile Acids Are Related to Cardiometabolic Risk Factors in Young Adults. J. Clin. Endocrinol. Metab..

[51] Oizel K., Chauvin C., Oliver L., Gratas C., Geraldo F., Jarry U., Scotet E., Rabe M., Alves-Guerra M.C., Teusan R. (2017). Efficient Mitochondrial Glutamine Targeting Prevails Over Glioblastoma Metabolic Plasticity. Clin. Cancer Res..

[52] Chen J., Cui L., Lu S., Xu S. (2024). Amino acid metabolism in tumor biology and therapy. Cell Death Dis..

[53] Miska J., Chandel N.S. (2023). Targeting fatty acid metabolism in glioblastoma. J. Clin. Investig..

[54] Zhou W., Yao Y., Scott A.J., Wilder-Romans K., Dresser J.J., Werner C.K., Sun H., Pratt D., Sajjakulnukit P., Zhao S.G. (2020). Purine metabolism regulates DNA repair and therapy resistance in glioblastoma. Nat. Commun..

[55] Jiang N., Xie B., Xiao W., Fan M., Xu S., Duan Y., Hamsafar Y., Evans A.C., Huang J., Zhou W. (2022). Fatty acid oxidation fuels glioblastoma radioresistance with CD47-mediated immune evasion. Nat. Commun..

[56] Djurovic Z., Jovanovic V., Obrenovic R., Djurovic B., Soldatovic I., Vranic A., Jakovljevic V., Djuric D., Zivkovic V. (2020). The importance of the blood levels of homocysteine, folate and vitamin B12 in patients with primary malignant brain tumors. JBUON.

[57] Baxter M.E., Miller H.A., Chen J., Williams B.J., Frieboes H.B. (2023). Metabolomic differentiation of tumor core versus edge in glioma. Neurosurg. Focus.

[58] Tasci E., Jagasia S., Zhuge Y., Camphausen K., Krauze A.V. (2023). GradWise: A novel application of a rank-based weighted hybrid filter and embedded feature selection method for glioma grading with clinical and molecular characteristics. Cancers.

[59] Mao W., Zhou J., Hu J., Zhao K., Fu Z., Wang J., Mao K. (2022). A pan-cancer analysis of FAT atypical cadherin 4 (FAT4) in human tumors. Front. Public Health.

[60] Nakata S., Yuan M., Rubens J.A., Kahlert U.D., Maciaczyk J., Raabe E.H., Eberhart C.G. (2021). BCOR Internal Tandem Duplication Expression in Neural Stem Cells Promotes Growth, Invasion, and Expression of PRC2 Targets. Int. J. Mol. Sci..

[61] Donson A.M., Amani V., Griesinger A.M., Calzadilla A., Grimaldo E., Willard N., Foreman N.K., Mulcahy-Levy J. (2024). Etmr-22. Identification of 5-Flurouracil As a Selective Therapy in a Novel EP300::Bcor Fusion Glioma Tumor Model. Neuro-Oncol..

[62] Krämer A., Green J., Pollard J., Tugendreich S. (2014). Causal analysis approaches in Ingenuity Pathway Analysis. Bioinformatics.

[63] Tasci E., Jagasia S., Zhuge Y., Sproull M., Cooley Zgela T., Mackey M., Camphausen K., Krauze A.V. (2023). RadWise: A Rank-Based Hybrid Feature Weighting and Selection Method for Proteomic Categorization of Chemoirradiation in Patients with Glioblastoma. Cancers.

[64] Guyon I., Elisseeff A. (2003). An introduction to variable and feature selection. J. Mach. Learn. Res..

[65] Voting Classifier. https://scikit-learn.org/stable/modules/generated/sklearn.ensemble.VotingClassifier.html.

