# Supplementary material for: GLIO-Select: Machine Learning-Based Feature Selection and Weighting of Tissue and Serum Proteomic and Metabolomic Data Uncovers Sex Differences in Glioblastoma
Source: Int J Mol Sci. 2025 May 2;26(9):4339. doi: 10.3390/ijms26094339 (PMC12072282; doi:10.3390/ijms26094339)
Supplement: Supplementary file 1 [file ijms-26-04339-s001.zip › ijms-3522541-Supplementary Materials/ijms-3522541-Supplementary Materials.pdf]

Supplementary Materials

Supplementary Table S1. Selected feature names list for all datasets

Supplementary Table S2. The effects of feature selection methods on accuracy rate (%) for the local preCRT-based proteomic dataset.

ML-ACC	Before FS	LASSO	mRMR
SVM	67.879	93.550	98.139
LR	89.827	92.641	97.230
KNN	76.926	88.918	97.230
RF	86.277	95.411	96.364
AdaBoost	94.459	90.779	92.641
Voting	89.827	93.550	96.321

Supplementary Table S3. The effects of feature selection methods on accuracy rate (%) for CPTAC proteomic dataset.

ML-ACC	Before FS	LASSO	mRMR
SVM	59.526	97.000	95.947
LR	75.737	97.000	97.000
KNN	59.737	97.000	96.000
RF	69.684	99.000	98.000
AdaBoost	97.000	97.000	97.000
Voting	83.737	98.000	97.000

Supplementary Table S4. The effects of feature selection methods on accuracy rate (%) for the local level one preCRT-based metabolomic dataset.

ML-ACC	Before FS	LASSO	mRMR
SVM	68.225	77.533	72.814
LR	70.000	77.532	70.043
KNN	57.749	70.000	70.043
RF	70.044	79.394	74.675
AdaBoost	71.905	75.801	69.134
Voting	71.775	77.532	70.996

Supplementary Table S5. The effects of feature selection methods on accuracy rate (%) for CPTAC metabolomic dataset.

ML-ACC	Before FS	LASSO	mRMR
SVM	54.666	60.000	61.334
LR	60.000	60.000	61.334
KNN	64.000	60.000	62.667
RF	62.667	58.667	64.000
AdaBoost	56.000	57.333	58.667

**Voting**	65.333	64.000	65.333

Supplementary Table S6. The effects of feature selection methods on accuracy rate (%) for the TCGA-GBM/UCI ML Repository molecular dataset.

**ML-ACC**	**Before FS**	**LASSO**	**mRMR**
**SVM**	55.980	59.095	60.511
**LR**	61.086	58.809	60.233
**KNN**	56.539	52.245	49.980
**RF**	59.090	58.809	60.511
**AdaBoost**	59.388	57.952	60.519
**Voting**	59.650	60.217	61.070

Supplemental Table S7. The metabolomic features identified in the serum NCI NIH and the CPTAC tissue based datasets. Green fill indicates known association with biological sex based on existing literature and use in the clinic and/or association with glioma or more specifically in some cases glioblastoma (GBM)

Compound	Compound explanation/Metabolic role	Documented association with biological sex	Documented association with glioma	Role in sex differences and or glioma
**NCI NIH Serum Metabolomic Dataset**				
GLU-THR	dipeptide formed from L-threonine and L-glutamic acid residues	no	yes	sex differences for this dipeptide are understudied; there is a connection to glioma based on connection between glioma and amino acid metabolism
N-FORMYLGLYCINE	lysosomal activity	no	no	none yet directly identified
2,3-DIHYDROXYISOVALERATE	amino acid biosynthesis	no	no	none yet directly identified
PEG n5	Polyethylene Glycol	no	no	possible contaminant
TESTOSTERONE SULFATE	male sex hormone	yes	yes	detected in both men and women, higher in men, possible relationship to androgen deficiency and connection to glioma prognosis
GUANOSINE	purine nucleoside	no	yes	may play a role in amino acid metabolism
HOMOCYSTEINE	amino acid	yes	yes	higher in men, evolving role in GBM metabolism
GLYCEROL	compounds, role in gluconeogenesis and lipolysis	yes	no	generally higher in women
TRIMETHYLLYSINE	post-translationally modified amino	no	no	carnitine biosynthesis and epigenetic modifications
XANTHOSINE	purine nucleoside	yes	yes	role in purine metabolism
CAFFEINE	stimulant	no	no	limited clinical relevance to glioma based on current literature
LYSOPE (18)	lysophospholipid	no	no	limited clinical relevance to glioma based on current literature
THROMBOXANE B2	eicosanoid invoved in platelet aggregation	yes	yes	higher in men, implicated in angiogenesis in GBM
N-ACETYLGLYCINE +Na	amino acid derivative	no	no	limited clinical relevance to glioma based on current literature, associated with fat loss
6-CARBOXYHEXANOATE+Na	hexanoic acid, medium chain fatty acid	no	yes	associated with fatty acid metabolism
ALLOCHOLIC ACID+FA	bile acid derivative	yes	no	higher in females
**CPTAC Tissue Metabolomic Dataset**				
PANTOTHENIC ACID	vitamin B5, precursor to coenzyme A (CoA)	no	no	none yet directly identified, metabolic connections in breast cancer
3-HYDROXYBUTYRIC ACID	ketone body increased during fasting	no	no	none yet directly identified, metabolic connections to ketogenic approaches
SCYLLO-INOSITOL	naturally occuring cyclo hexanol	no	no	none yet directly identified, metabolic connections but not in GBM
HOMOCYSTEINE	amino acid	yes	yes	higher in men, evolving role in GBM metabolism
N-METHYLALANINE	amino acid	yes	no	higher in females, no direct relationship to glioma
D-MANNITOL	sugar alcohol, also administered to decrease intracranial pressure	no	yes	detected in pre and post operative samples in GBM
O-PHOSPHOCOLAMINE	phospholipid metabolism	no	yes	compound understudied for sex differences, lipid metabolism relevant in GBM
L-2-AMINOADIPIC ACID	metabolite of lysine, stuctural analog of glutamine	no	yes	compound understudied for sex differences, glutamine and lipid metabolism relevant in GBM
D-GLUCONIC ACID	oxidised form of glucose	no	yes	precursor to glycosaminoglycans which are implicated in GBM
GALACTITOL	sugar alcohol	no	yes	none yet directly identified, metabolic connections to GBM via Leloir cycle (galactose scavenging/remodelling)
TIMONACIC	amino acid derivative	no	no	none yet directly identified
D (+) GALACTOSE	simple sugar	no	no	none yet directly identified
GLYCEROL 3-PHOSPHATE	glycerophospholipid, intermediate	yes	yes	sex differences based on adipose tissue and fatty acid
